# Isolated Mast Cell–Mediated Angioedema: Clinically Different but Endotypically Similar to Chronic Spontaneous Urticaria

**DOI:** 10.1111/cea.70214

**Published:** 2026-01-11

**Authors:** Thomas Buttgereit, Claudia Hayford, Carolina Vera Ayala, Sherezade Moñino‐Romero, Yi‐Kui Xiang, Pavel Kolkhir, Carolin Steinert, Denise Freier, Markus Magerl, Jörg Scheffel, Karsten Weller

**Affiliations:** ^1^ Institute of Allergology, Charité—Universitätsmedizin Berlin, Corporate Member of Freie Universität Berlin and Humboldt‐Universität zu Berlin Berlin Germany; ^2^ Fraunhofer Institute for Translational Medicine and Pharmacology ITMP Immunology and Allergology Berlin Germany

**Keywords:** angioedema, antihistamines, basophil histamine release, CSU, IgE, IL‐24, mast cell, omalizumab, TPO, urticaria

## Abstract

- CSU AE and CSU W+AE patients show similar rates of autoimmune and autoallergic endotypes.
- The study supports the current CSU definition and recommendations in the global urticaria guideline.

To the Editor,

Chronic spontaneous urticaria (CSU) is the most frequent form of chronic urticaria defined by the spontaneous occurrence of wheals (W), angioedema (AE), or both (W+AE) for more than 6 weeks [1]. At least two autoimmune endotypes have been described in the pathophysiology of CSU: type I autoimmunity (autoallergy) CSU patients have IgE autoantibodies against autoallergens, while in type IIb autoimmune CSU, patients additionally have functional IgG autoantibodies that activate mast cells through binding to FcεRI and IgE [2, 3].

A debate has emerged whether patients with standalone angioedema, that is, AE patients, should be considered a phenotype of CSU or a different disease entity. Recent findings showed phenotype and endotype‐related differences in AE patients compared to W+AE patients, that is, higher age at disease onset, higher mean basophil counts, negative basophil activation test, lower total‐IgE levels and the absence of antibodies against FcεRI [4, 5, 6, 7, 8].

In this single‐center study at the Urticaria Center of Reference and Excellence in Berlin, we compared AE (*n* = 39) and W+AE (*n* = 32) patients regarding their clinical data and a selection of recommended tests/biomarkers to screen for underlying type of autoimmunity including response to treatment (complete: > 90% improvement, partial: < 90% improvement and no response: unchanged) [9]. Patients with hereditary angioedema due to C1 inhibitor deficiency were used as controls (EA4/078/17).

AE patients were older (64% female, median 58 years, range 22–78, *p* < 0.05) than W+AE patients (71% female, median 50 years, range 21–80) and showed significantly (*p* < 0.05) higher Angioedema Control Test Scores (median, 13, range 0–16) than W+AE patients (median, 7, range 0–16), that is, had better disease control. Moreover, AE patients showed numerically lower values in the Angioedema Quality of Life Questionnaire (median, 37, range 3–77, *n* = 26) than W+AE patients (median, 46, range 0–88, *n* = 27), that is, had a clinically significantly reduced disease burden. W+AE patients reported longer attack duration (median, 24 h, range 4–120) than AE patients (median, 12 h, range 1–120). Overall, angioedema occurred primarily at the face involving lips, eyelids and tongue. However, W+AE patients significantly more often reported swellings of the hands and feet than AE patients (71% vs. 41% and 64% vs. 35%, respectively, *p* < 0.05 each). In AE patients, angioedema occurred significantly more frequently on the tongue for the first time (34% vs. 11%, *p* < 0.05), with W+AE patients more frequently reporting the feet as the first location (30% vs. 9%, *p* < 0.05). A history of NSAID‐induced angioedema was found in 10% (*n* = 4) of AE and in 6% (*n* = 2) of W+AE patients. At baseline, basophil and eosinophil counts did not differ between AE and W+AE patients and there were no statistical differences observed in the rates of positive Basophil Histamine Release Assay BHRA (BHRA+, 38% vs. 25%), although they were higher than in the control group (HAE‐C1INH, 14%) (Figure 1A). Furthermore, there were no differences in basophil counts between BRHA+ and BHRA negative (BHRA‐) patients in both groups. BHRA+ patients, however, showed lower eosinophil counts compared to their BHRA‐ counterparts in all groups, which was statistically significant (*p* < 0.005) in AE patients (Figure 1B). The mean ± SD total IgE values did not differ between AE and W+AE patients (257 ± 451 ng/mL vs. 258 ± 313 ng/mL) but were significantly (*p* < 0.05) higher than in controls. Total IgE levels were low (< 96 ng/mL) in 28% and 26% of AE and W+AE patients and the mean total IgE levels in both AE and W+AE patients were numerically lower in BHRA+ patients than BHRA− patients (Online Repository). Among AE and W+AE patients with low total IgE, 64% (*n* = 7) and 25% (*n* = 2) were BHRA+, respectively. Mean levels of IgE anti‐IL‐24 and IgE anti‐TPO (thyroid peroxidase) as well as serum soluble FcεRI (sFcεRI) did not differ in AE and W+AE patients (Online Repository), also not when stratified by BHRA result. At baseline, 90% and 100% of AE and W+AE patients used treatment for their disease. Of these, 83% (*n* = 29) AE patients and 90% (*n* = 28) W+AE patients used prophylactic treatment with antihistamines AH (any dose) and 17% (*n* = 6) and 10% (*n* = 3) used AH on‐demand. In *n* = 28 AE and *n* = 16 W+AE patients, response to prophylactic treatment with AH (standard dose and up to 4‐fold dose) were assessed in routine clinical care for a period of (mean ± SD) 13 ± 22 and 10 ± 11 months, respectively. 7%, 37% and 26% of AE patients and 25%, 10% and 65% of W+AE patients showed complete, partial and no response, respectively. Out of AE and W+AE patients using AH prophylactically, 17% (*n* = 5) and 29% (*n* = 8), respectively, additionally used omalizumab for prophylactic treatment. Treatment response to omalizumab could be assessed in 10 AE patients and 15 W+AE patients for a period of 24 ± 21 and 10 ± 8 months, respectively; and 83%, 17% and 0% of AE patients and 77%, 8% and 15% of W+AE showed complete, partial and no response, respectively. In both AE and W+AE patients, higher total IgE levels (> 96 ng/mL) and BHRA− at baseline were associated with better response to omalizumab.

**FIGURE 1:**
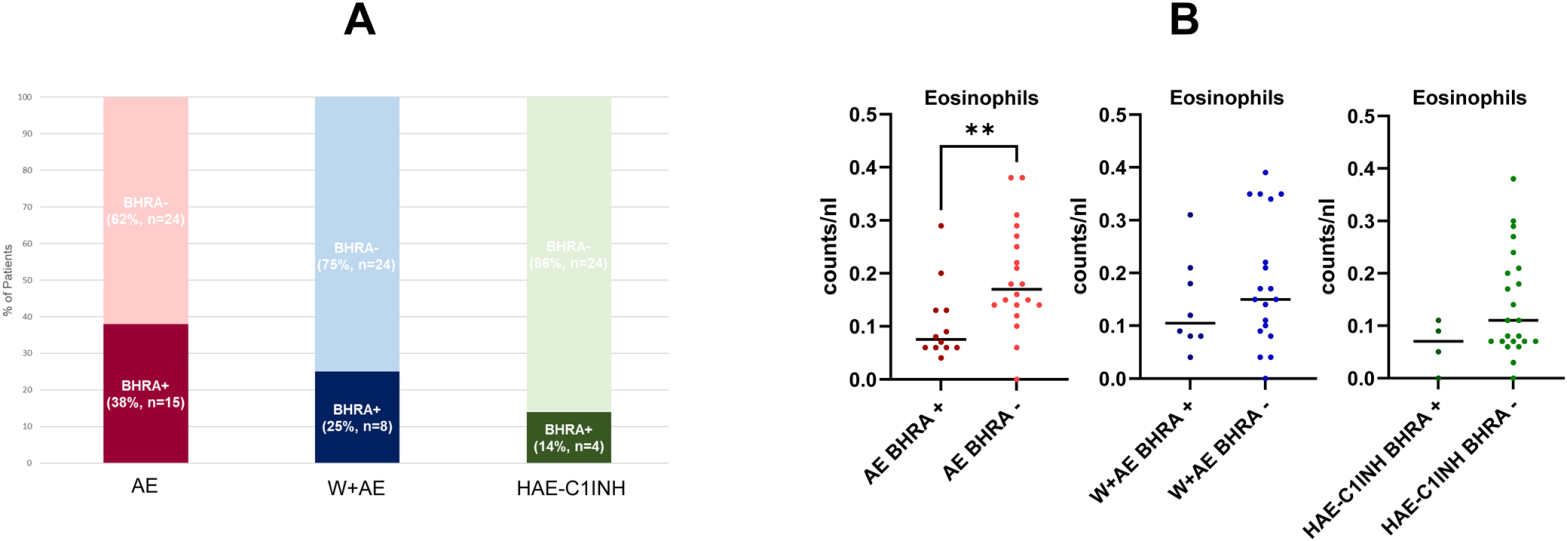
(A) Concentration of Basophil Histamine Release Assay (BHRA) was measured and a cut‐off 23.9 ng/mL was used to divide in BHRA− (below 23.9 ng/mL) or BHRA+ (above 23.9 ng/mL). Frequency of BHRA− or BHRA+ patients within chronic spontaneous urticaria (CSU) groups with standalone angioedema (AE) or wheals (W) and AE (W+AE) are summarised and plotted in a Histogram. Patients with hereditary angioedema (HAE) due to C1 inhibitor (C1INH) deficiency (HAE‐C1INH) were controls. Cumulative percentage was calculated by dividing the cumulative frequency by the total number of observations (*n*), then multiplying it by 100. (B) Eosinophil counts were plotted over BHRA+ and BHRA− classification in the AE, W+AE and controls (HAE‐C1INH). Mann–Whitney *p* value was calculated. Significance was shown as **p ≤ 0.01.

Due to the small sample size as a limitation of the study, the results regarding response to treatment should be interpreted with caution. Furthermore, not all known predictive markers for underlying type of autoimmunity in CSU were investigated. Not least, there is no guarantee that no wheals will occur in the future in the AE patients studied.

In conclusion, the results of this study add evidence that AE patients are more similar than different to W+AE patients in terms of predictive markers for the underlying type of autoimmunity supporting the notion they belong to the same entity.

## Author Contributions

Karsten Weller, Thomas Buttgereit, and Markus Magerl designed the study. Claudia Hayford contributed to the statistical analysis. Sherezade Moñino‐Romero, Carolin Steinert, Carolina Vera Ayala, Yi‐Kui Xiang, and Jörg Scheffel contributed to the development of immunoassays and cellular assays. Yi‐Kui Xiang, Sherezade Moñino‐Romero, Carolina Vera Ayala, and Carolin Steinert performed the laboratory tests. Carolina Vera Ayala, Thomas Buttgereit, Denise Freier, and Markus Magerl contributed to patient recruitment and interpreted the data. Thomas Buttgereit prepared the first version of the paper. All authors contributed critical input and approved the submitted version.

## Funding

This work was supported, in part, by the International Center for Angioedema Research (icare) at Charité.

## Consent

This study was approved by the local ethics committee (EA4/078/17) at Charité—Universitätsmedizin Berlin. Patients provided written informed consent before any assessment was performed.

## Conflicts of Interest

T. Buttgereit declares no conflicts of interest in relation to this work. Outside of it, he is/was a speaker and/or advisor for and/or received research funding from Almirall, Aquestive, AstraZeneca, BioCryst, CSL‐Behring, Galderma, GSK, Hexal, KalVista, Medac, Novartis, Otsuka, Pharming, Pharvaris, Roche, Sanofi, Swixx BioPharma, and Takeda. C. Hayford has no conflicts of interest to declare. C.V. Ayala is a clinical trial/registry investigator for BioCryst, CSL Behring, Intellia, Ionis Pharmaceuticals, Novartis, Pharvaris, Jasper, KalVista Pharmaceuticals, Blueprint, Celldex, incyte, and Takeda. S. Moñino‐Romero has no conflicts of interest to declare. Yi‐K. Xiang has no conflicts of interest to declare. P. Kolkhir was a speaker/consultant for BioCryst, Merus, Novartis, Roche, and ValenzaBio outside of the submitted work. C. Steinert has no conflicts of interest to declare. D. Freier has no conflicts of interest to declare. M. Magerl has received personal fees/non‐financial support from Astria, BioCryst, CSL Behring, Inois, Intellia, KalVista Pharmaceuticals, Octapharma, Pharming, Pharvaris, and Shire Takeda. J. Scheffel has no conflict of interest associated with this study. J.S. has conducted studies for, received research funds/was an advisor for Allakos, Ascilion, Aquestive, AstraZeneca, Beiersdorf, CSL Behring, Celldex, Escient, Evommune, Genentech, Invea, Novartis, Sanofi, Servier, Septerna, Third Harmonic Bio, ThirdRock, ThirdHarmonic, and ThermoFisher. K. Weller reports grants from Novartis and Takeda outside the submitted work and personal fees from CSL Behring, Novartis, Moxie, and Takeda outside the submitted work.

## Data Availability

The data that support the findings of this study are available from the corresponding author upon reasonable request.
